# Virtual microscope interface to high resolution histological images

**DOI:** 10.1186/1746-1596-3-S1-S10

**Published:** 2008-07-15

**Authors:** Josef Feit, Luděk Matyska, Vladimír Ulman, Lukáš Hejtmánek, Hana Jedličková, Marta Ježová, Mojmír Moulis, Věra Feitová

**Affiliations:** 1 Institute of Pathology, Masaryk University, Brno, Czech Republic; 2 Faculty of Informatics, Masaryk University, Brno, Czech Republic; 3 Dept. of Dermatovenerology,, St. Anna Hospital, Masaryk University, Brno, Czech Republic; 4 Dept. of Radiology, St. Anna Hospital, Masaryk University, Brno, Czech Republic

## Abstract

The Hypertext atlas of Dermatopathology, the Atlas of Fetal and Neonatal Pathology and Hypertext atlas of Pathology (this one in Czech only) are available at . These atlases offer many clinical, macroscopic and microscopic images, together with short introductory texts. Most of the images are annotated and arrows pointing to the important parts of the image can be activated.

The Virtual Microscope interface is used for the access to the histological images obtained in high resolution using automated microscope and image stitching, possibly in more focusing planes. Parts of the image prepared in advance are downloaded on demand to save the memory of the user's computer. The virtual microscope is programmed in JavaScript only, works in Firefox/Mozilla and MSIE browsers without need to install any additional software.

## Introduction

The Hypertext Atlas of Dermatopathology [1] is available on the Internet since 2008. It contains 4840 clinical, macroscopic and histologic images. Recently the Atlas of Fetal and Neonatal Pathology is available as well. The Atlas of Pathology for pre-graduate students of medicine is available as well (in Czech only) and new atlases are under preparation (today with about 2300 images).

The atlases contain annotated images (arrows pointing to important parts of the images can be activated) and short introductory texts. Histological images are taken using motorized microscope to take image parts, which are later stitched together. The image stitching is based on analysis of overlapping parts of individual image tiles and joined together using the gradient running on randomly generated curve to obtain the best cosmetic results.

## Methods

### Hardware, image acquisition

Leica DMLA microscope with a set of PlanApo lenses (HC Fluotar 5/0.15, HC PlanApo 10/0.30, 20/0.50, 40/0.70, 100/1.35 and a Plan 2/0.07 lenses) equipped with the Nikon DMX-1200 digital camera is used to obtain image parts at the resolution at 1200 × 1020 pixels, 3 × 8 bit colour. Motorized stage (Merzhäuser) is automatically moved from one image to another. The system is controlled by Lucia DI (LIM, Prague). Home made software is used to create composed, very large pictures and to prepare the virtual microscope image stacks.

### Source texts of the atlas

The atlas source texts are in XML data format. Programs (written mostly in Perl 5.8 programming language) are used to parsing and checking the document structure and to generate the final HTML files, which are uploaded on the server. The overall size of the atlases is about 110 GB of data.

### Image post-processing, virtual microscope

Each image part can be taken in one or more focusing levels. This image stack can be processed by pan-focusing function, which selects sharp areas of each image from the stack to create one image tile and overcoming the problem of image artefacts caused by uneven slides. This feature is especially useful if only slides of suboptimal quality are available (slides of rare cases, from old slide collections etc.). We use usually 3 levels, their distance of focusing planes varies according to the objective used.

Alternatively the whole image stacks are taken and saved. The resulting images are created from stitching tiles from each plane separately. This approach allows creation of multilevel images, which can be focused. This approach we use especially in images taken in high resolution using 100× immersion lens, as in bone marrow biopsies. We use usually 5 or 7 focusing planes, sometimes more (up to 13).

After stitching each image is digitally manipulated (colour correction, sharpening), archived and cut into pieces, side of which are of multiples 256. Larger pieces 512 and more) are converted into 256 parts. All these image parts are saved into structured directories.

The browser loads proper parts of image according to current viewport, magnification and focusing level. It reacts to user's actions (image dragging, magnification changing, change in the size of the virtual microscope window, focusing) through catching events, calculates the names of corresponding new image tiles, which are loaded and added into the DOM of the image being displayed. The image parts, which got out of he viewport, are released from memory.

The individual parts can be stored on the server or locally on the disc. No special server application is needed. The magnification can be changed, images can be dragged and the images saved in stacks can be focused. This approach is used especially in images t Users can change the size of the virtual microscope window up to the full screen.

## Results

Our atlases are available at . The access requires registration, which is free. In January 2008 about 1450 users were registered. Combined size of all the atlases is over 110 GB of data.

The virtual microscope interface works reasonably smooth. This approach does not require installation of any additional software (but the JavaScript in the browser must be enabled). MSIE since the version 4 and Mozilla family of Internet browsers are supported.

**Figure 1:**
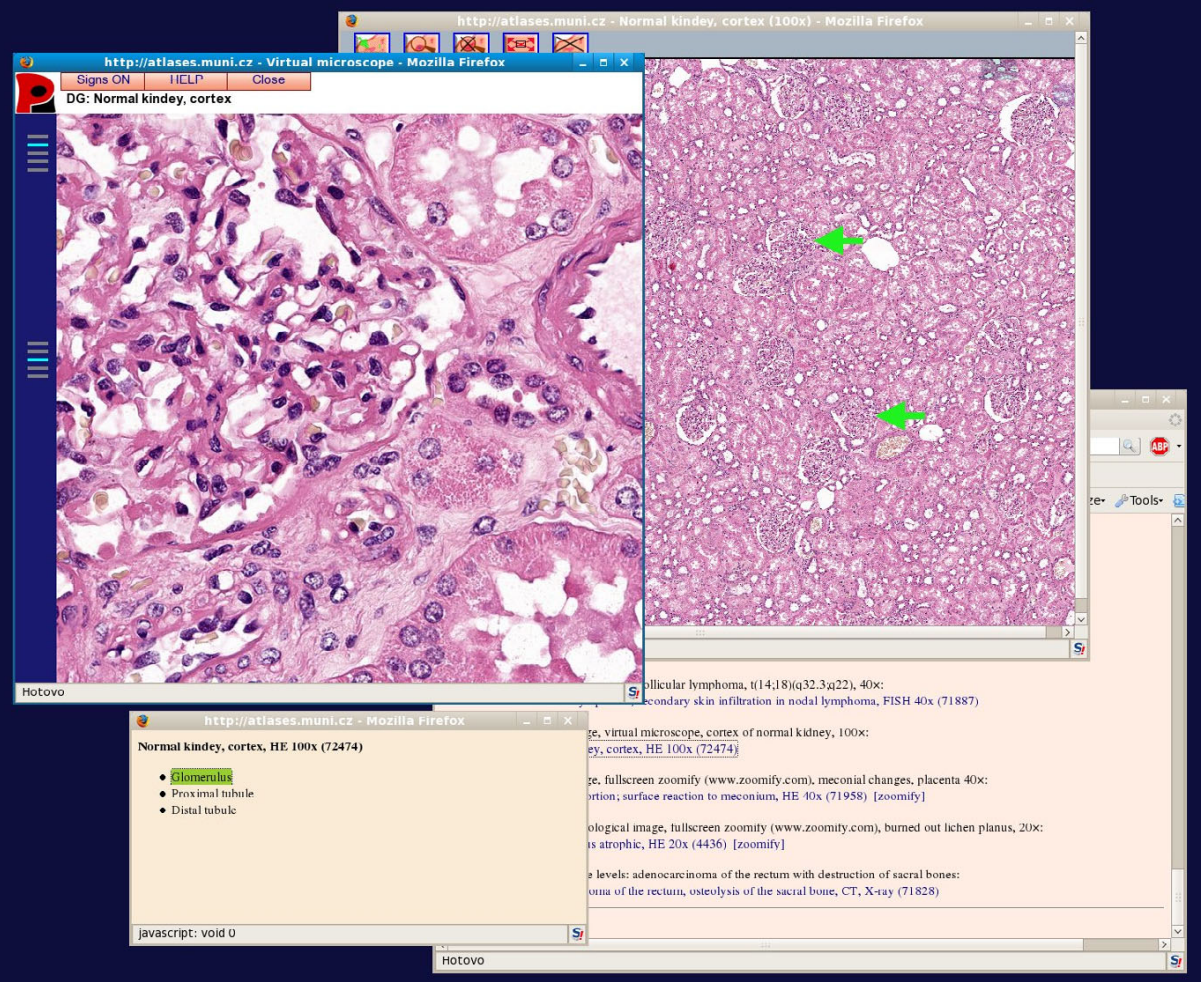
The text window is in the background, lower left is the list of signs, and the arrows are activated in the basic window; virtual microscope window is in upper left.

The interface consists of several windows: the text of the atlas, the window with an image in basic (900 px) size with possible activation of arrows, list of signs and window with the virtual microscope with magnification and focus changer (see the Figure 1). Users can open more images at the same time for easy comparing.

## Discussion

Publishing teaching materials in the Internet has many advantages. In properly designed publication systems the complexity does not grow with extent of the source texts and images. It is easy to publish new version, to add new materials or reflect comments and desires of the students. The quality of the images is very high, usually much better than in printed textbooks and their number is virtually unlimited. Moreover, virtual microscope offers new experience to the students (dragging, focusing, magnification changing), leading to more active approach to learning [2]. Virtual slides can capture whole tissue specimens, not only selected areas ("negative" areas are important as well) and can be used in quizzes as well. In our teaching labs we do not use microscopes any more. Digital slides do not worn out, can be easily replaced if new, more instructive case is available and can be accessed any time from anywhere. Virtual microscope is suitable for preparing diagnostic seminars and reference collections. One slide is enough to prepare the case, so even small specimens can be used for seminar without the danger of cutting through the tissue without having representative specimen for each student or participant.

## Conclusion

Our atlases are continuously upgraded and expanded. In addition to the above-mentioned Atlas of Dermatopathology and Atlas of Fetopathology and Neonatal Pathology we are preparing new atlases (of muscle pathology and bone marrow biopsy). In future image sharing of our images will be possible as well, so that other teachers will be able to include links to images in our atlases, comment them according to their taste and still have access to all the features of the virtual microscope.
